# Supplementary material for: Dynamics of HIV-1 Gag Processing as Revealed by Fluorescence Lifetime Imaging Microscopy and Single Virus Tracking
Source: Viruses. 2022 Feb 8;14(2):340. doi: 10.3390/v14020340 (PMC8874525; doi:10.3390/v14020340)
Supplement: Supplementary file 1 [file viruses-14-00340-s001.zip › viruses-1580696-supplementary.pdf]

Supplementary Materials

Dynamics of HIV-1 Gag Processing as Revealed by Fluorescence Lifetime Imaging Microscopy and Single Virus Tracking

Chen Qian, Annica Flemming, Barbara Müller and Don C. Lamb

This file includes:

Text S1: Description of the phasor approach to analyzing FLIM data.

Figure S1: Analysis of the processing status of Gag.eCFP and Gag.ieCFP by immunoblot.

Figure S2: Effect of ratio of wild-type to eCFP-labelled Gag on eCFP fluorescence lifetime in VLPs.

Figure S3: Effect of temperature on eCFP fluorescence lifetime in VLPs.

Figure S4: Effect of the matrix domain on the fluorescence lifetime of eCFP.

Figure S5: Coexpression of Gag and Gag.ieCFP in HeLa Kyoto cells verified by immunoblot.

Figure S6: Mean intensity and lifetime of assembly traces that did not show a lifetime decrease corresponding to the intensity increase.

Figure S7: Additional examples of particles showing eCFP lifetime changes indicative of maturation.

Table S1: Summary of particle traces used for analysis.

Table S2: Additional single virus tracing analysis using wavelet tracking method.

Other supporting information for this manuscript include the following:

Movie S1: Movie of maturing VLP shown in Figure 4A. (.mp4)

Movie S2: Movie of maturing VLP shown in Figure S6A. (.mp4)

Movie S3: Movie of maturing VLP shown in Figure S6B. (.mp4)

Movie S4: Movie of maturing VLP shown in Figure S6C. (.mp4)

Text S1. Description of the phasor approach to analyzing FLIM data.

A phasor plot is a graphical way of analyzing fluorescence lifetime decays [1,2]. Each lifetime measurement can be represented as a vector. The Cartesian coordinates s and g of the phasor (a contraction of the term phase vector) represent, respectively, the first sine and cosine Fourier coefficients of the signal:

$$s_{i,j}(\omega) = \frac{1}{M_{Inst}} \cdot \int_0^{2\pi} I_{i,j}(t) \cdot \sin(\omega t - \Phi_{Inst}) dt \Big/ \int_0^{2\pi} I_{i,j}(t) dt \qquad (1)$$

$$g_{i,j}(\omega) = \frac{1}{M_{Inst}} \cdot \int_0^{2\pi} I_{i,j}(t) \cdot \cos(\omega t - \Phi_{IRF}) dt \Big/ \int_0^{2\pi} I_{i,j}(t) dt \qquad (2)$$

where the indices i and j define the coordinates of the pixel on the image, $I_{i,j}(t)$ represents the photon counts at time bin t. The modulation frequency, ω, corresponds to $2\pi/T$ where T is the full time range of the lifetime decay histogram (here T = 40 ns). Φ_{Inst} and M_{Inst} are the correction factors for the phase shift and demodulation caused by the instrument response function. These were calculated using a reference sample with a known mono-exponential lifetime (Atto 425, 3.6 ns, Atto-Tec).

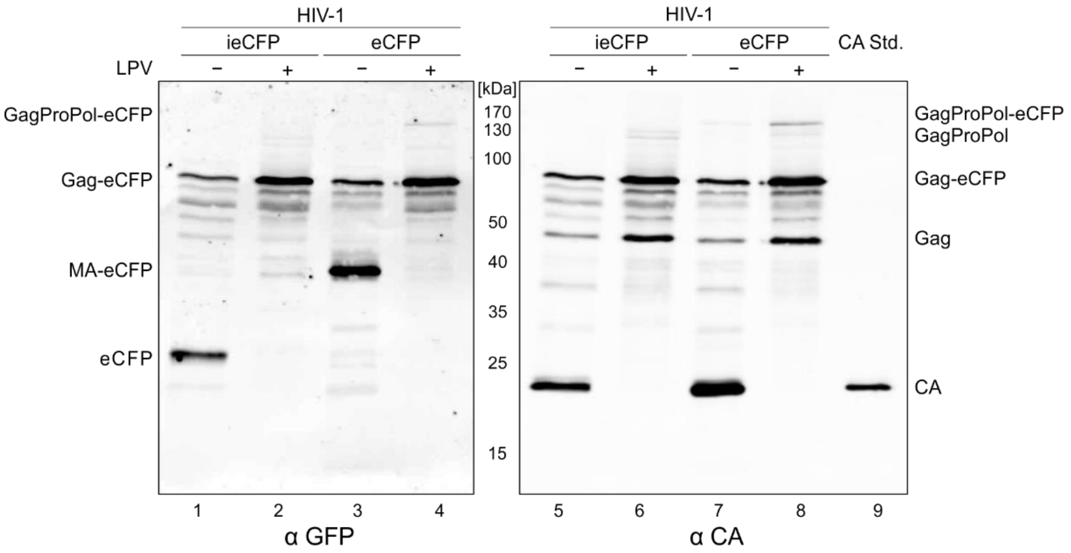

Figure S1. Analysis of the processing status of Gag.eCFP and Gag.ieCFP by immunoblot. Immunoblot of purified HIV^{eCFP} and HIV^{ieCFP} VLPs from the supernatant of HEK293T cells that were co-transfected with pCHIV and either $pCHIV^{ieCFP}$ or $pCHIV^{eCFP}$ in a 1:1 molar ratio and subsequently grown in the absence or presence of 2 µM LPV. Supernatants were harvested at 48 hpt and concentrated via ultracentrifugation through a 20% sucrose cushion. Samples were separated by SDS-PAGE and analyzed by immunoblot using polyclonal rabbit antiserum raised against eGFP and HIV-1 CA. Bound antibody was detected by quantitative immunoblot using a Li-Cor Odyssey infrared scanner.

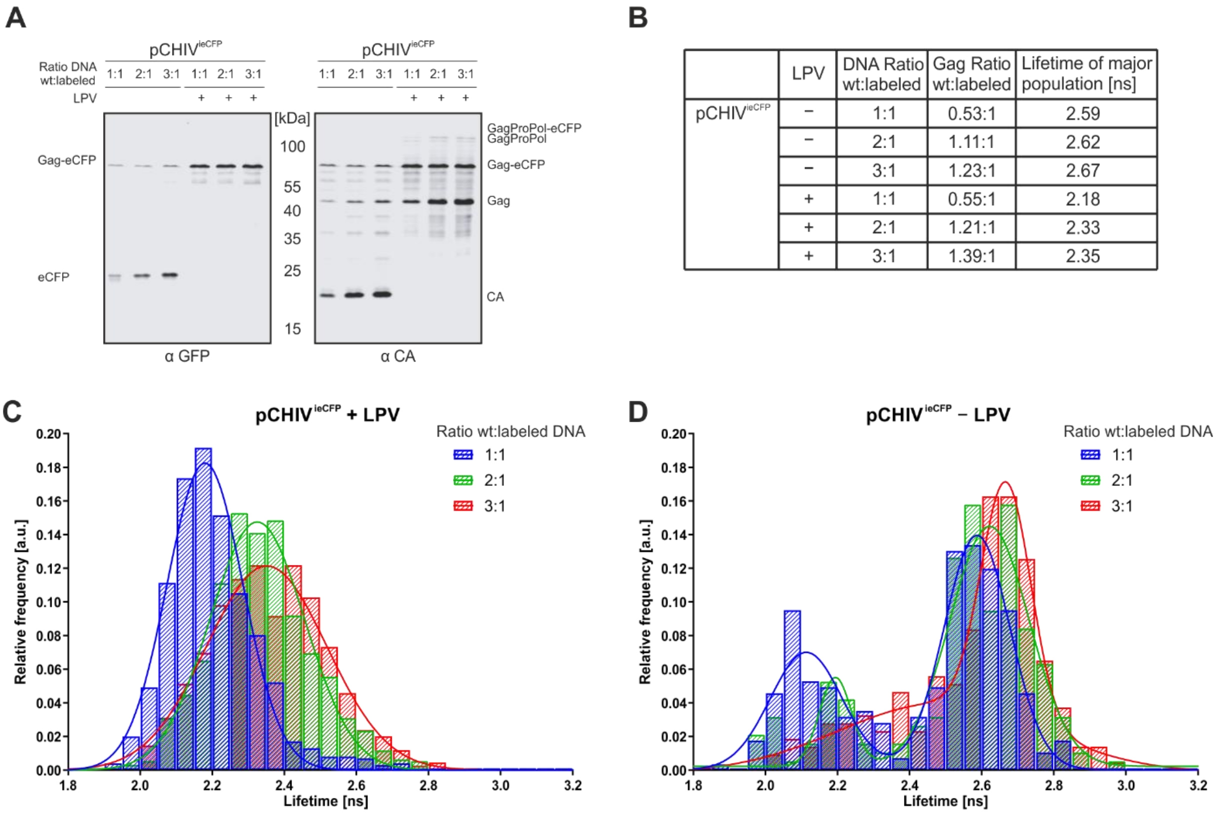

	LPV	DNA Ratio wt:labeled	Gag Ratio wt:labeled	Lifetime of major population [ns]
pCHIV^ieCFP	−	1:1	0.53:1	2.59
	−	2:1	1.11:1	2.62
	−	3:1	1.23:1	2.67
	+	1:1	0.55:1	2.18
	+	2:1	1.21:1	2.33
	+	3:1	1.39:1	2.35

Figure S2. Effect of ratio of wild-type to eCFP-labelled Gag on eCFP fluorescence lifetime in VLPs. (**A**) Immunoblot of VLPs produced by co-transfecting 293T cells at specified molar ratios of wt:labelled DNA. Cells were incubated in the absence or presence of 2 µM LPV. Supernatants were harvested at 48 hpt and analyzed by immunoblot. (**B**) Table of results showing the ratio of wt:labelled protein and the eCFP lifetime of the major particle population produced by each corresponding DNA ratio. Protein ratios were quantified from the immunoblot in A. eCFP lifetimes were extracted from the histograms in (C) and (D). (**C**, **D**) Histograms of particle lifetimes extracted using phasor analysis. Each distribution was fitted with a Gaussian distribution function with up to 2 populations (solid line).

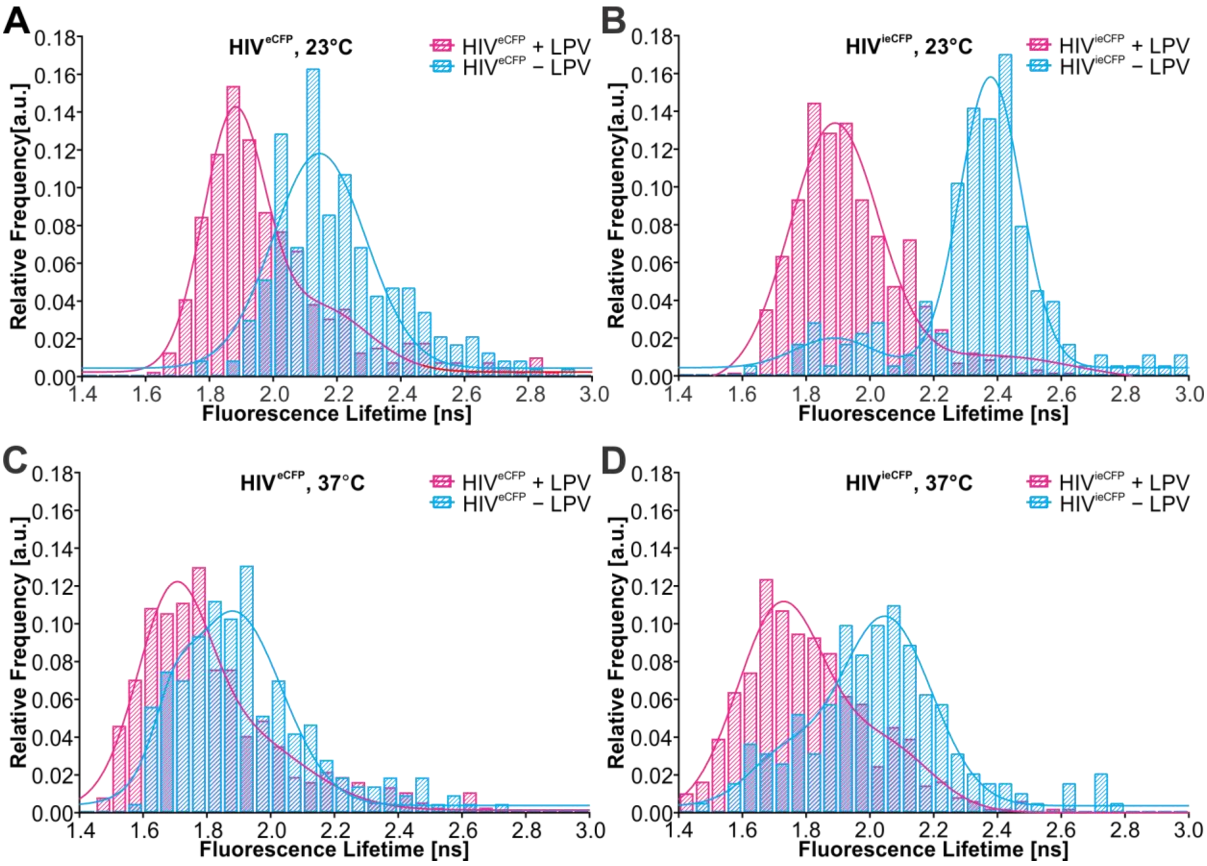

Figure S3. Effect of temperature on the eCFP fluorescence lifetime in VLPs. The fluorescence lifetimes of immature (*cyan*) and mature (*magenta*) purified particles were measured at 23°C and 37°C. Fluorescence lifetime histograms of (**A**) HIV^{eCFP} at 23°C (n_{-LPV} = 237; n_{+LPV} = 392), (**B**) HIV^{ieCFP} at 23°C (n_{-LPV} = 176, n_{+LPV} = 567), (**C**) HIV^{eCFP} at 37°C (n_{-LPV} = 214, n_{+LPV} = 370) and (**D**) HIV^{ieCFP} at 37°C (n_{-LPV} = 191, n_{+LPV} = 485). Lines show the fit of the lifetime distributions to the sum of two Gaussians.

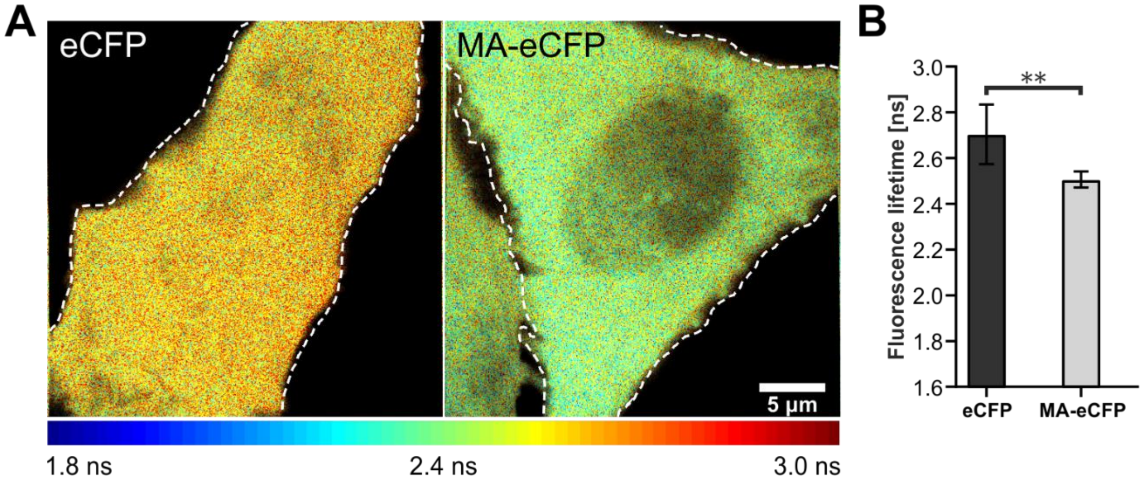

Figure S4. Effect of the matrix domain on fluorescence lifetime of eCFP. (**A**) Fluorescence lifetime images of cytoplasmic eCFP and MA-eCFP. (**B**) Mean and SD of fluorescence lifetime of cytoplasmic eCFP and MA-eCFP. *n* = 7 cells for eCFP) and 10 cells for MA-eCFP. The images are colored according to the 'jet' colormap with a range of 1.8 ns - 3.0 ns. Scale bars: 5 µm. Statistical analysis was performed using a Welch's t-test (**: $p < 0.01$).

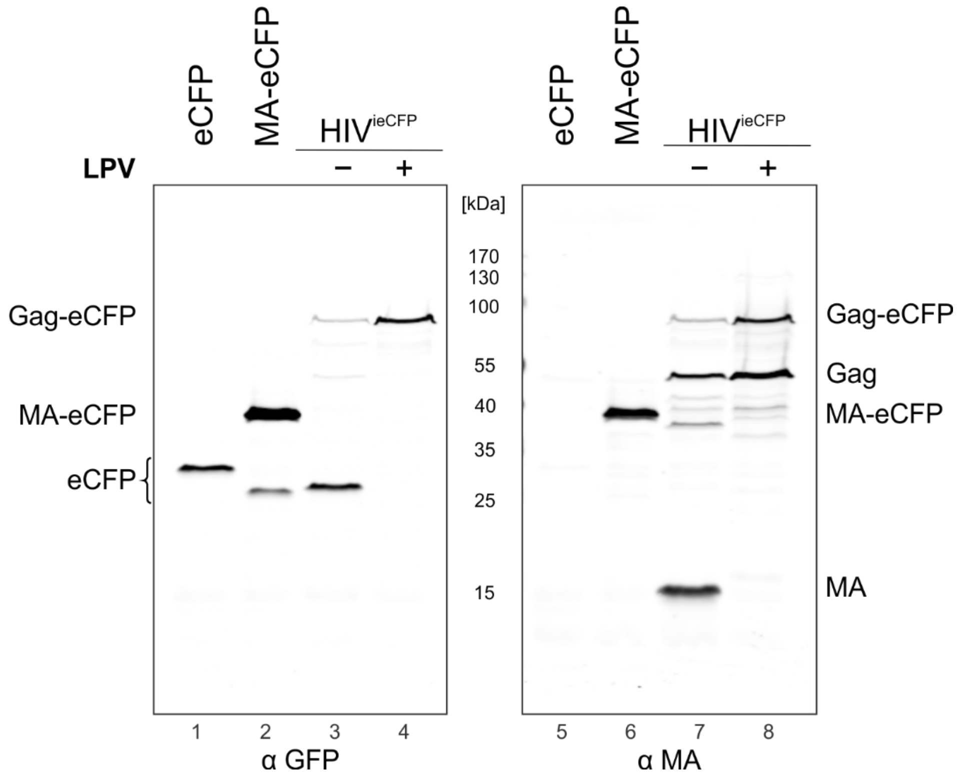

Figure S5. Coexpression of Gag and Gag.ieCFP in HeLa Kyoto cells verified by immunoblot. Lysates of HeLa Kyoto cells transiently expressing the indicated constructs were separated by SDS-PAGE and analyzed by detected by quantitative immunoblot (Li-Cor) using the indicated antisera. For HIV^{ieCFP}, cells were co-transfected with pCHIV and $pCHIV^{ieCFP}$ in a 1:1 molar ratio. Molecular masses of standard proteins in kDa are indicated in the middle.

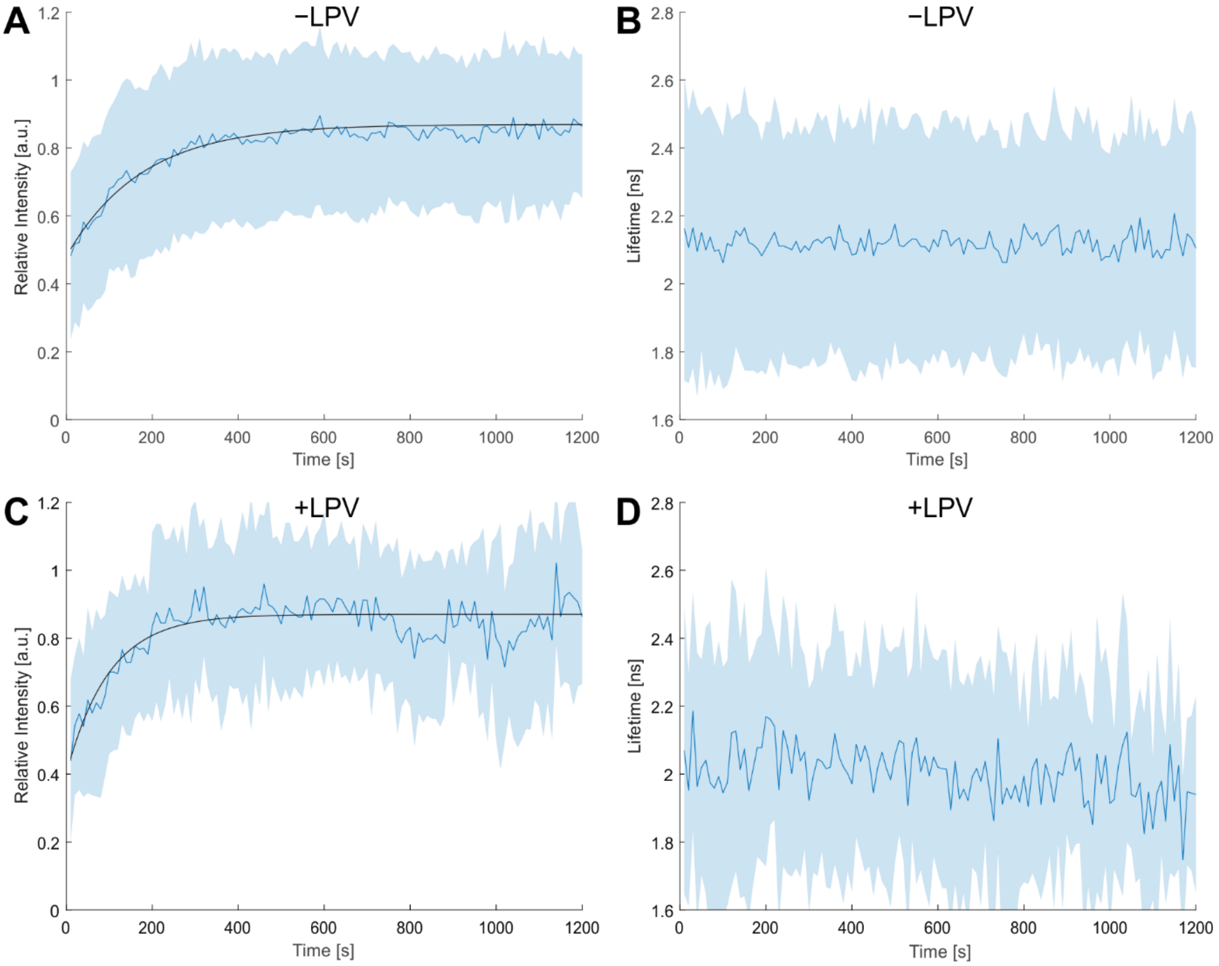

Figure S6: Mean intensity and lifetime of assembly traces that did not show a lifetime decrease corresponding to the intensity increase. (**A**) Mean fluorescence intensity and (**B**) mean fluorescence lifetime of assembly sites of pCHIV^{ieCFP} transfected HeLa Kyoto cells which did not show a lifetime decrease corresponding to the intensity increase. n = 184 assembly sites from 4 cells. (**C**) Mean fluorescence intensity and (**D**) mean fluorescence lifetime of similar assembly sites in cells treated with LPV. n = 35 from 2 cells. Mean fluorescence intensity of the traces were fitted with a saturating exponential function (black line). The fitted rate constant was 5.7 (± 0.6) × 10^{-3} s^{-1} in (A) and 10.1 (± 3.1) × 10^{-3} s^{-1} in (C).

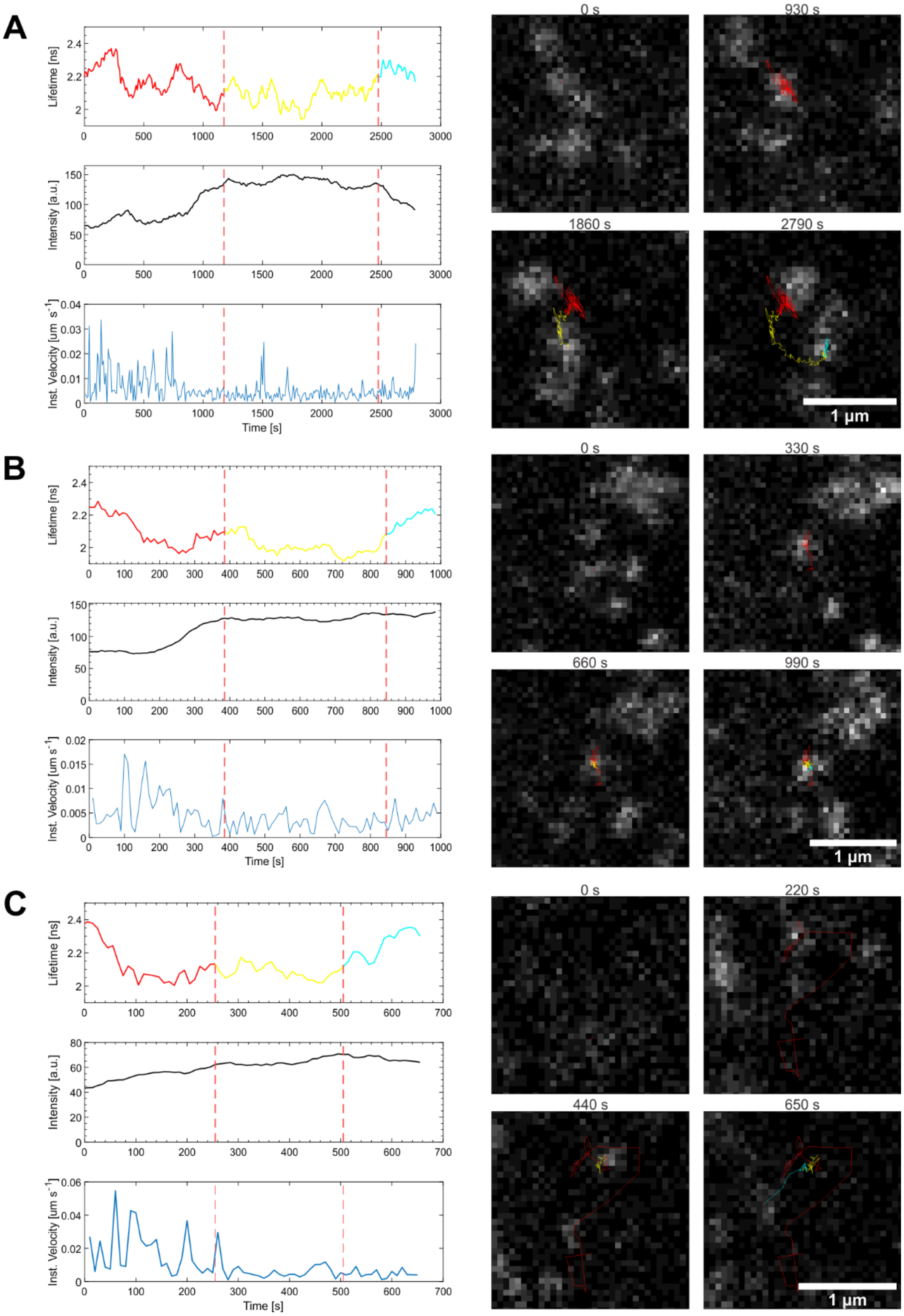

Figure S7: Additional examples of particles showing eCFP lifetime changes indicative of maturation. (**A-C**) Lifetime, intensity and velocity plots of particles showing lifetime changes indicative of maturation following assembly. The lifetime plots are colored by the identifiable state of the particle (red: assembly, yellow: plateau phase, blue: maturation). Images of the tracked particles recorded at the indicated times are shown on the right. Scale bars: 1 µm.

Table S1: Summary of particle traces used for analysis

Particle Type	Total Tracks	Assembly (Intensity)	Assembly (Intensity and Lifetime)	Gag processing
pCHIV^{ieCFP} − LPV	489	354	170	11
pCHIV^{ieCFP} + LPV	105	86	51	0

Table S2. Additional single virus tracing analysis using wavelet tracking method.

Particle Type	Total cells	Total Tracks	Gag processing
pCHIV^{ieCFP} −LPV	6	1992	46
pCHIV^{ieCFP} +LPV	4	1693	0

To verify that the lifetime changes we observed occurred exclusively on non-LPV-treated cells, consistent with being indicative of Gag processing, we repeated our single virus tracing analysis using a wavelet transformation algorithm [3,4], implemented directly in PAM. The algorithm performs an *à trous* wavelet decomposition on the image to denoise and enhance the fluorescence signal from VLPs before detection by thresholding. We performed the tracking with relatively lenient filtering and then manually reviewed each trace to look for lifetime changes indicative of Gag processing. Additional cells were also included in the analysis.

Supplementary References

1. Digman, M.A.; Caiolfa, V.R.; Zamai, M.; Gratton, E. The phasor approach to fluorescence lifetime imaging analysis. *Biophys. J.* **2008**, *94*, L14-16, doi:10.1529/biophysj.107.120154.
2. Ranjit, S.; Malacrida, L.; Jameson, D.M.; Gratton, E. Fit-free analysis of fluorescence lifetime imaging data using the phasor approach. *Nat. Protoc.* **2018**, *13*, 1979-2004, doi:10.1038/s41596-018-0026-5.
3. Olivo-Marin, J.C. Extraction of spots in biological images using multiscale products. *Pattern Recognit.* **2002**, *35*, 1989-1996, doi:10.1016/S0031-3203(01)00127-3.
4. Messer, P.K.; Henß, A.-K.; Lamb, D.C.; Wintterlin, J. A Multiscale Wavelet Algorithm for Atom Tracking in STM Movies. *New J. Phys.* **2022**, *(In Press)*.
